# Combination Strategies to Enhance the Efficacy of Antimicrobial Peptides against Bacterial Biofilms

**DOI:** 10.3389/fmicb.2017.02409

**Published:** 2017-12-07

**Authors:** Lucia Grassi, Giuseppantonio Maisetta, Semih Esin, Giovanna Batoni

**Affiliations:** Department of Translational Research and New Technologies in Medicine and Surgery, University of Pisa, Pisa, Italy

**Keywords:** antimicrobial peptides, bacterial biofilms, combination therapies, antibiofilm strategies, synergistic interactions

## Abstract

The great clinical significance of biofilm-associated infections and their inherent recalcitrance to antibiotic treatment urgently demand the development of novel antibiofilm strategies. In this regard, antimicrobial peptides (AMPs) are increasingly recognized as a promising template for the development of antibiofilm drugs. Indeed, owing to their main mechanism of action, which relies on the permeabilization of bacterial membranes, AMPs exhibit a strong antimicrobial activity also against multidrug-resistant bacteria and slow-growing or dormant biofilm-forming cells and are less prone to induce resistance compared to current antibiotics. Furthermore, the antimicrobial potency of AMPs can be highly increased by combining them with conventional (antibiotics) as well as unconventional bioactive molecules. Combination treatments appear particularly attractive in the case of biofilms since the heterogeneous nature of these microbial communities requires to target cells in different metabolic states (e.g., actively growing cells, dormant cells) and environmental conditions (e.g., acidic pH, lack of oxygen or nutrients). Therefore, the combination of different bioactive molecules acting against distinct biofilm components has the potential to facilitate biofilm control and/or eradication. The aim of this review is to highlight the most promising combination strategies developed so far to enhance the therapeutic potential of AMPs against bacterial biofilms. The rationale behind and beneficial outcomes of using AMPs in combination with conventional antibiotics, compounds capable of disaggregating the extracellular matrix, inhibitors of signaling pathways involved in biofilm formation (i.e., quorum sensing), and other peptide-based molecules will be presented and discussed.

## Introduction

Over the last years, growing efforts have been devoted to the identification of novel therapeutic strategies capable of coping with biofilm-associated infections. Indeed, bacteria organized in biofilm display a dramatically reduced susceptibility (up to 1000 times) to conventional antibiotics compared to their planktonic counterparts, causing a high rate of treatment failure and persistence of many types of infections (e.g., lung infections in cystic fibrosis patients, wound infections, biomaterial-associated infections) (Parsek and Singh, 2003). Several mechanisms are responsible for the enhanced antibiotic-tolerance of biofilms including: (i) reduced diffusion or sequestration of antimicrobials through the biofilm extracellular matrix; (ii) presence of slow-growing and even dormant cells (“persisters”) highly refractory to the action of antibiotics targeting bacterial metabolism; (iii) exchange of mobile genetic elements encoding resistance determinants due to cell vicinity (Høiby et al., 2010; Lebeaux et al., 2014).

**FIGURE 1:**
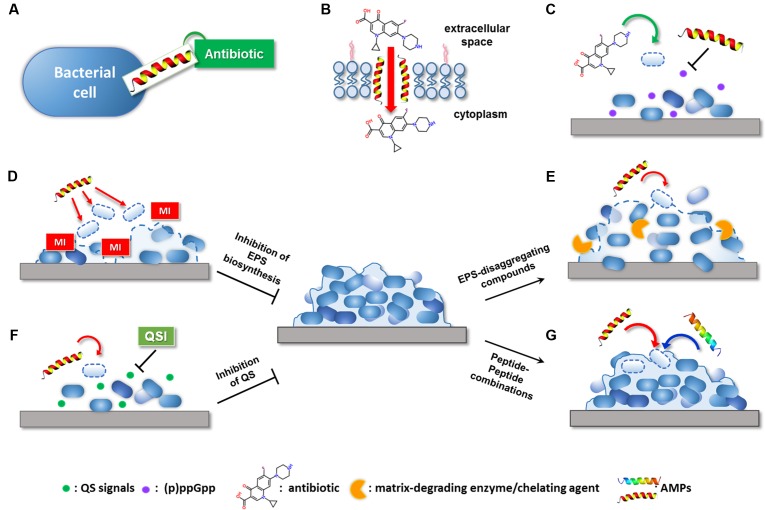
Possible mechanisms of the synergistic activity of AMP-based combinations against bacterial biofilms. AMPs can potentiate the antibiofilm effect of conventional antibiotic by: **(A)** extending antibiotic spectrum of action; **(B)** promoting antibiotic intracellular uptake through membrane destabilization; **(C)** interfering with signaling molecules involved in biofilm formation. Compounds able to target the biofilm EPS can potentiate AMP activity by: **(D)** inhibiting matrix production in forming biofilms; **(E)** causing matrix disaggregation in preformed biofilms. **(F)** QSIs can facilitate the killing of early surface-colonizing bacteria by AMPs by interfering with signaling molecules implicated in biofilm formation. **(G)** AMPs may synergize with other AMPs with mechanisms still largely unknown. MI, matrix inhibitor; QSI, quorum sensing inhibitor. Dashed lines indicate killing of bacteria or inhibition/disaggregation of biofilm matrix.

The use of AMPs as novel antibiofilm agents holds considerable promise and represents an increasingly explored research area (Di Luca et al., 2015). As substantial components of the innate immunity, AMPs are widely distributed throughout the microbial, animal, and plant kingdoms. The majority of AMPs are short (12–50 amino acids), cationic, and amphipathic molecules endowed with a bacterial membrane-perturbing mechanism of action. Due to the low specificity of their molecular target, AMPs exhibit a broad-spectrum of activity, reduced propensity to induce resistance and high potential to target metabolically dormant cells (Batoni et al., 2016; Grassi et al., 2017a). Several studies have demonstrated the ability of AMPs to interfere with various stages of biofilm formation by preventing the initial adhesion of bacterial cells to surfaces, by targeting planktonic cells before they enter into the biofilm structure or by destroying mature biofilms through the detachment and/or killing of biofilm-embedded bacteria (Segev-Zarko et al., 2015). Many tested AMPs have been shown to be more effective in inhibiting the early phases of biofilm development than in eradicating established biofilms. This is partially due to the multiple interactions that such molecules may establish with components of the extracellular matrix that surrounds and protects cells in mature biofilms (e.g., DNA, polysaccharides, proteins) (Batoni et al., 2016). To improve the antibiofilm properties of AMPs, current focus is on combining them with conventional antibiotics and/or other antimicrobial compounds (Pletzer et al., 2016; Walkenhorst, 2016). Indeed, the identification of synergistic (peptide-based) combinations has the potential to decrease the effective concentration of the active molecules and to extend their spectrum of action, thereby reducing possible toxic side effects and the spread of resistance, often linked to monotherapy regimens (Walkenhorst, 2016). In addition to these general advantages, the use of combination therapies seems to be particularly indicated in the case of biofilms as their complex architecture requires to target cells in different metabolic states and environmental conditions (Batoni et al., 2016). The present article provides a global overview of the most encouraging AMP-based combinations against biofilms and the possible mechanisms of the synergistic action. Particular attention is focused on combinatorial strategies involving the use of AMPs with: (i) conventional antibiotics, (ii) compounds capable of disaggregating the biofilm extracellular matrix or inhibiting its synthesis, (iii) inhibitors of QS and/or other signaling pathways implicated in biofilm formation, and (iv) other AMPs and/or peptide-based molecules. Examples of combinations involving the use of AMPs in conjunction with other compounds against bacterial biofilms are reported in **Table 1**, while putative mechanisms of synergism are illustrated in **Figure 1**.

**Table 1:** Antibiofilm combination strategies involving AMPs or peptide-based molecules.

Peptide(s)	Combined compound(s)	Bacterial specie(s)	Proposed mechanism	Reference
BMAP-28	Vancomycin	*Enterococcus faecalis, Staphylococcus aureus*	AMP-mediated uptake	Orlando et al., 2008
CRAMP	Vancomycin	*Salmonella enterica* serovar Typhimurium	AMP-mediated uptake	Mishra et al., 2015
G10KHc	Tobramycin	*Pseudomonas aeruginosa*	AMP-mediated uptake	Eckert et al., 2006
DJK-5 and DJK-6	Ciprofloxacin, ceftazidime, tobramycin	*Acinetobacter baumannii, Klebsiella pneumoniae, Escherichia coli, Pseudomonas aeruginosa*	Degradation of (p)ppGpp	de la Fuente-Nuñez et al., 2015
IDR-1018	Ciprofloxacin	*Pseudomonas aeruginosa*	Degradation of (p)ppGpp	Reffuveille et al., 2014
AMP38	Imipenem	*Pseudomonas aeruginosa*	–	Rudilla et al., 2016
DJK-6	Imipenem, meropenem	*Klebsiella pneumoniae*	–	Ribeiro et al., 2015
HPMA	Ciprofloxacin	*Acinetobacter baumannii*	–	Gopal et al., 2014
Lactoferrin	Ciprofloxacin	*Porphyromonas gingivalis*	–	Wakabayashi et al., 2009
Nisin	Penicillin	*Enterococcus faecalis*	–	Tong et al., 2014
Tachyplesin III	Piperacillin/tazobactam	*Pseudomonas aeruginosa*	–	Minardi et al., 2007
Nisin	DHBA	*Staphylococcus aureus*	Inhibition of PIA synthesis	Ahire and Dicks, 2015
Temporin 1Tb	L-cysteine	*Staphylococcus epidermidis*	Inhibition of PIA synthesis	Maisetta et al., 2016
Human β-defensin-3	DNase I	*Haemophilus influenzae*	Matrix degradation	Jones et al., 2013
KSL-W	Dispersin B	*Acinetobacter baumanii, Klebsiella pneumoniae, Staphylococcus aureus, Staphylococcus epidermidis*	Matrix degradation	Gawande et al., 2014
TN-5	Alginate lyase	*Pseudomonas aeruginosa*	Matrix degradation	Bahar et al., 2015
Temporin 1Tb	EDTA	*Staphylococcus epidermidis*	Matrix destabilization	Maisetta et al., 2016
TB_KKG6A and TB_L1FK	EDTA	*Pseudomonas aeruginosa, Staphylococcus aureus*	Matrix destabilization; cell wall perturbation	Grassi et al., 2017b
Human β-defensin-2	Nitric oxide	*Pseudomonas aeruginosa*	Biofilm dispersal	Ren et al., 2016
Daptomycin	FS3	*Staphylococcus aureus*	Inhibition of quorum sensing	Cirioni et al., 2013
DD_13_	RNA III-inhibiting peptide	*Staphylococcus aureus, Staphylococcus epidermidis*	Inhibition of quorum sensing	Balaban et al., 2004
Gramicidin S	Polymyxin B (PMB)	*Pseudomonas aeruginosa*	PMB-mediated translocation through the outer membrane	Berditsch et al., 2015
Citropin 1.1, temporin A, analog of tachyplesin I	Colistin	*Pseudomonas aeruginosa, Staphylococcus aureus*	–	Jorge et al., 2017
Nisin	Colistin, polymyxin B	*Pseudomonas aeruginosa*	–	Field et al., 2016

## Combination of AMPs with Conventional Antibiotics

A large body of evidence highlighted the beneficial effect of using AMPs in conjunction with conventional antibiotics, often leading to enhanced activity against multidrug-resistant strains and expanded spectrum of action of antibiotics (Maisetta et al., 2009; Wakabayashi et al., 2009; Gopal et al., 2014; Mishra et al., 2015; Ribeiro et al., 2015; Rudilla et al., 2016) (**Table 1**).

### Promotion of Antibiotic Uptake by AMPs

Mishra et al. (2015) have recently succeeded in extending the spectrum of vancomycin toward Gram-negative bacteria through the covalent linkage of the antibiotic with a cathelicidin-related antimicrobial peptides (CRAMP). Due to its ability to translocate across the outer membrane, it is likely that CRAMP functioned as a carrier peptide for the transfer of vancomycin into the periplasm of Gram-negative bacteria (**Figure 1A**). Beyond this peculiar synergistic effect, it is commonly recognized that perturbation of bacterial membranes caused by AMPs plays a key role in enhancing the intracellular uptake and efficacy of numerous antibiotics (Dosler and Mataraci, 2013; Mohamed et al., 2016) (**Figure 1B**). In this regard, co-administration of tobramycin and the chimeric peptide G10KHc has proved effective against biofilms of *Pseudomonas aeruginosa*, resulting in a nearly 10,000-fold increase in the bactericidal activity of the antibiotic. G10KHc was demonstrated to mediate internalization of a small-molecule dye (propidium iodide) providing strong evidence that sublethal doses of the peptide could promote the uptake of small molecules, such as tobramycin, into *P. aeruginosa* cells by inducing membrane damage (Eckert et al., 2006). Similarly, the enhancing effect of the cathelicidin BMAP-28 on vancomycin activity observed against Gram-positive cocci has been attributed to the increased access of the antibiotic through the cytoplasmic membrane. Interestingly, the use of peptide-coated ureteral stents in combination with intraperitoneal vancomycin resulted in reduced biofilm formation by *Staphylococcus aureus* and *Enterococcus faecalis* in a rat model of urinary infection, suggesting that AMP-based combinations may represent new opportunities for the prevention of implant-associated infections (Orlando et al., 2008).

### AMP Potentiation of Antibiotics by Interfering with Signaling Pathways Involved in Biofilm Formation

In addition to facilitating antibiotic uptake, some AMPs have been found to potentiate the antibiofilm activity of currently available antibiotics by interfering with signaling molecules that are involved in biofilm formation and maintenance (**Figure 1C**). In this regard, de la Fuente-Nuñez et al. (2014, 2015) have designed three optimized antibiofilm peptides (i.e., IDR-1018, DJK-5, and DJK-6) capable of degrading the stress-related signaling nucleotide (p)ppGpp. The effect of such peptides on (p)ppGpp levels substantially increased the ability of several antibiotics to inhibit biofilm formation and treat mature biofilms formed by multidrug-resistant pathogens, reducing the effective antibiotic concentrations up to 64 times (Reffuveille et al., 2014; de la Fuente-Nuñez et al., 2015). Surprisingly, the antibiofilm peptide DJK-6 displayed strong synergy with carbapenems also against biofilms of carbapenemase-producing *Klebsiella pneumoniae*, highlighting the usefulness of AMPs in repurposing conventional antibiotics (Ribeiro et al., 2015).

## Combination of AMPs with Anti-Matrix Compounds

Biofilm bacteria are enclosed in a self-produced extracellular matrix constituted by a complex mixture of extracellular polymeric substances (EPS). EPS components, such as exopolysaccharides, exoproteins, and extracellular DNA (eDNA), mediate cell-to-cell and cell-to-surface connections, playing a crucial role in biofilm formation and stabilization. In addition, they create a physical barrier that protects biofilm cells against host immune system and antimicrobial agents (Flemming and Wingender, 2010). Electrostatic repulsion and sequestration by matrix polymers have been demonstrated to decrease the bioavailability of AMPs and to reduce their antibiofilm potential (Batoni et al., 2016). Nevertheless, several studies have succeeded in potentiating the antibiofilm activity of AMPs by combining them with compounds capable of inhibiting the synthesis of EPS components in forming biofilms (**Table 1** and **Figure 1D**) and/or promoting matrix disaggregation in preformed biofilms (**Table 1** and **Figure 1E**) (Fleming and Rumbaugh, 2017). Specific examples of these combinations are provided in the following paragraphs.

### Combination of AMPs with Matrix-Inhibiting Compounds

Inhibition of extracellular matrix biosynthesis may positively influence the antibiofilm effect of AMPs by favoring their interaction with bacterial cells prior to bacterial incorporation into the protective EPS (**Figure 1D**).

#### Sulfhydryl Compounds

Treatment of forming biofilms of *S. aureus* with sulfhydryl compounds (e.g., dithiothreitol, β-mercaptoethanol, L-cysteine) has proved to reduce the production of the PIA, a major component of staphylococcal EPS involved in intercellular aggregation during biofilm formation. The proposed mechanism of action was the downregulation of the *ica* operon that encodes essential enzymes for PIA biosynthesis (Wu et al., 2011). The matrix-inhibiting effect of L-cysteine has been recently exploited to enhance the efficacy of the frog skin-derived peptide temporin 1Tb (1Tb) against forming biofilms of *Staphylococcus epidermidis*. The combination demonstrated a striking ability to reduce the total biofilm biomass of a PIA-positive strain of *S. epidermidis* at levels much higher than L-cysteine or 1Tb used alone (Maisetta et al., 2016).

#### Iron Chelators

Iron chelators have also been reported to prevent biofilm formation by staphylococci by reducing PIA biosynthesis (Lin et al., 2012). Chelation of iron by 2,3-dihydroxybenzoic acid (DHBA) has been demonstrated to prevent *S. aureus* from forming a stable biofilm and to promote the bactericidal activity of the bacteriocin nisin against planktonic cells prior to their aggregation. Furthermore, incorporation of nisin and DHBA into polymeric nanofibers has proved to be a suitable approach to ensure a long-lasting inhibitory effect against *S. aureus* and prevent chronic wound infections (Ahire and Dicks, 2015).

### Combination of AMPs with Matrix-Disaggregating Compounds

Dispersal of preformed biofilms by matrix-disaggregating compounds represents another valuable approach to facilitate the targeting of biofilm-associated bacteria by AMPs (**Figure 1E**). Combining matrix disassembly with the bactericidal action of AMPs has the potential not only to facilitate the killing of biofilm-detaching cells but also to avoid their dispersal to other sites with consequent risk of secondary or systemic infections.

#### Matrix-Degrading Enzymes

Various classes of matrix-degrading enzymes (e.g., proteases, deoxyribonucleases, glycoside hydrolases) have displayed a remarkable ability to disperse preformed biofilms of multiple bacterial species (Chaignon et al., 2007; Kaplan, 2010). The use of DNAse I has been reported to enhance the ability of the human β-defensin-3 (hBD-3) both in preventing biofilm formation of non-typeable *Haemophilus influenzae* and in killing biofilm-associated cells, highlighting the effectiveness of the enzyme in favoring peptide diffusion through the disassembled matrix (Jones et al., 2013). Analogously, degradation of matrix polysaccharides with dispersin B, a β-*N*-acetyl-glucosaminidase produced by the periodontal pathogen *Aggregatibacter actinomycetemcomitans*, has been shown to enhance the susceptibility of biofilm-forming bacteria to the synthetic peptide KSL-W. When formulated as a wound gel, the combination peptide-dispersin B exhibited strong synergism against mature biofilms of methicillin-resistant *S. aureus*, *S. epidermidis*, *K. pneumonia*, and *Acinetobacter baumannii*, suggesting its possible application in the treatment of chronic wound infections (Gawande et al., 2014).

#### Chelating Agents

Another dispersal strategy consists in the use of molecules capable of physically destabilizing the biofilm matrix structure, such as the chelating agent EDTA (Lebeaux et al., 2015). In this regard, we have recently reported that the sequestration of matrix-stabilizing ions (e.g., magnesium, calcium) by EDTA is effective in disaggregating *S. epidermidis* biofilms, causing significant enhancement of the antibiofilm activity of 1Tb. Accordingly, when combined with EDTA, the peptide was able to eradicate mature biofilms formed *in vitro* on silicone catheters, indicating the potential use of such a combination in the lock therapy of colonized central venous access devices (Maisetta et al., 2016). In addition, due to the perturbing action of EDTA on the outer membrane of Gram-negative bacteria, peptide-EDTA combinations could also exert a direct synergistic effect on biofilm-embedded cells. For instance, the combination of some optimized analogs of 1Tb with EDTA resulted in a potentiated antibacterial activity against both planktonic cultures and biofilms of *P. aeruginosa* (Grassi et al., 2017b). Interestingly, EDTA was also able to markedly potentiate the activity of another recently described semi-synthetic AMP (lin-SB056-1) against mature biofilms of *P. aeruginosa*. Importantly, the peptide/EDTA combination almost completely inhibited the formation of biofilm-like structures (BLSs) in an artificial sputum medium closely resembling the complex environment found in the lung of cystic fibrosis patients, suggesting its possible employment in the treatment of *P. aeruginosa* infections (Maisetta et al., 2017).

#### Nitric Oxide

Exploiting natural dispersal signals to induce biofilm disassembly has also emerged as a promising route of investigation. The signaling molecule NO has been identified as a key mediator of biofilm dispersal controlling the transition from a sessile to a planktonic phenotype. In a wide range of bacterial species, NO-mediated dispersal has been associated with a decrease in the intracellular levels of the second messenger cyclic di-GMP (c-di-GMP), which leads to activation of EPS-degrading enzymes and bacterial motility (Barraud et al., 2015). Using a recently developed electrochemical NO-releasing catheter, Ren et al. (2016) have demonstrated a strong enhancing effect of physiological levels of NO on the activity of human β-defensin-2 (hBD-2) against preformed biofilms of *P. aeruginosa*.

## Combination of AMPs with Quorum Sensing Inhibitors

Bacteria coordinate gene expression in a cell density-dependent manner by producing and detecting small extracellular signaling molecules (autoinducers) in a process known as QS (Waters and Bassler, 2005). A variety of organic molecules have been associated with cell-to-cell communication, including AHLs in Gram-negative bacteria, oligopeptides in Gram-positive bacteria, and autoinducer-2 (AI-2) acting as universal interspecies signal. Detection of a threshold level of these molecules induces group-based behaviors in the bacterial population, among which are virulence factor production and biofilm formation (Davies et al., 1998). Considering its involvement in bacterial pathogenesis, inhibition of QS has emerged as a valuable approach to control infections without applying selective pressure for the development of resistance (Rasko and Sperandio, 2010). A large number of QSIs have been identified that can interfere with bacterial communication by preventing signal generation, degrading signaling molecules, and/or impeding signal reception (Kalia, 2013). The use of QSIs has been widely demonstrated to improve the antibiofilm activity of numerous conventional antibiotics both *in vitro* and *in vivo* (Brackman et al., 2011; Christensen et al., 2012). Balaban et al. (2004) have exploited the inhibitory activity of QSIs on biofilm formation to enhance the efficacy of a membrane-active dermaseptin derivative (DD_13_). Construction of a chimeric peptide composed of DD_13_ and the RNA III-inhibiting peptide (RIP), known to disrupt QS mechanisms in staphylococci, was reported to exert a potent antibiofilm activity in a rat graft infection model with methicillin-resistant *S. aureus* and *S. epidermidis*. Although both individual compounds were able to reduce biofilm formation, the chimeric construct (DD_13_-RIP) displayed higher efficiency in inhibiting staphylococcal colonization ensuring almost sterility at the lowest doses (Balaban et al., 2004). Coating of vascular grafts with FS3, a recently designed derivative of RIP (Baldassarre et al., 2013), has been shown to markedly increase the effect of the lipopeptide daptomycin administered intraperitoneally in a rat model of staphylococcal infection, confirming the relevance of QSIs as enhancers of peptide-based molecules (Cirioni et al., 2013) (**Table 1** and **Figure 1F**). Therefore, therapeutic strategies aimed at interfering not only with the viability of biofilm-associated cells but also with their virulence represent an encouraging solution to favor bacterial eradication by the host immune system and mitigate the outcome of the infection.

## Combination of AMPs with other AMPs or Peptide-Based Molecules

Natural AMP-based defense systems have evolved to act synergistically against microorganisms in the host environment. Synergism between AMPs has evolved as a natural strategy to ensure host protection against a broad-spectrum of pathogens, thereby explaining the presence of a wide array of AMPs within a single host (Yan and Hancock, 2001). Although the mechanisms underlying these synergistic interactions remain elusive, several studies have suggested that the organization of different AMPs in functional complexes may promote their cooperative antibacterial action. For example, the natural complex fly larvae immune peptides 7 (FLIP7) isolated from *Calliphora vicina* maggots has been shown to ensure a broad-spectrum antibiofilm activity due to the simultaneous presence of four distinct families of AMPs (i.e., defensins, cecropins, diptericins, and proline-rich peptides). Indeed, only the complex as a whole led to significant destruction of mature biofilms formed by both *Escherichia coli* and *S. aureus*, offering sub stantial advantages compared to a single-molecule approach (Gordya et al., 2017).

Despite prefiguring wide therapeutic opportunities, antibiofilm strategies involving the combination of different AMPs are just beginning to be explored (**Table 1** and **Figure 1G**). Berditsch et al. (2015) have recently defined a synergistic interaction between two cyclic membrane-active peptides, polymyxin B (PMB) and gramicidin S (GS), against both forming and mature biofilms of *P. aeruginosa* strain PAO1. Interaction of PMB with the LPS of Gram-negative bacteria has been proposed to facilitate the translocation of GS through the outer membrane of *P. aeruginosa*, enhancing the overall bactericidal effect against biofilm-forming cells. Similar synergistic and additive effects have been achieved by combining the polypeptide colistin with citropin 1.1, temporin A, and a linear analog of tachyplesin I. Although optimization of possible cytotoxic effects seems necessary, colistin-AMP combinations resulted to be effective also against double-species biofilms of *P. aeruginosa* and *S. aureus*, suggesting their potential for the treatment of polymicrobial biofilm-related infections (e.g., wound infections) (Jorge et al., 2017).

## Conclusion and Future Perspectives

Currently, the treatment of biofilm-associated infections represents a major challenge of modern medicine. Antibiotic therapy alone often fails in eradicating bacterial biofilms and generally requires high concentrations and/or repeated administrations of the drug, increasing the risk of adverse reactions and selection of resistance. The development of drug combinations involving the use of AMPs may represent a promising anti-biofilm strategy. Nevertheless, more research is needed for the in-depth evaluation of the synergistic properties of AMP-based combinations in order to take full advantage of their therapeutic potential. Indeed, despite the abundance of possible antibiofilm combinations, most synergism studies involving AMPs have been limited to the planktonic state (Jorge et al., 2012). Combinations of AMPs with lysostaphin (Desbois and Coote, 2011), bacteriophage-derived endolysins (Briers et al., 2014), and antimicrobial polymers (Piras et al., 2015), proved to be highly active against planktonic cells, represent promising candidates to be tested also against forming and mature biofilms. Furthermore, additional improvement and standardization of the methods used to determine synergism against biofilms are required to allow a reliable comparison of different combination strategies and a homogenous interpretation of the results. Standardized laboratory protocols along with the development of automated systems for data screening and processing may provide a more accurate understanding and prediction of synergistic interactions. Finally, progress needs to be made in validating *in vitro* studies in physiologically relevant biofilm model systems. Such studies will greatly contribute in translating peptide-based combinations into multifunctional antibiofilm drugs.

## Author Contributions

LG and GB wrote the manuscript. GM and SE critically edited the manuscript. All the authors gave the final approval.

## Conflict of Interest Statement

The authors declare that the research was conducted in the absence of any commercial or financial relationships that could be construed as a potential conflict of interest.

## Abbreviations

AHLs: *N*-acyl homoserine lactones
AI-2: autoinducer-2
AMPs: antimicrobial peptides
BLSs: biofilm-like structures
CRAMP: cathelicidin-related antimicrobial peptides
DHBA: 2,3-dihydroxybenzoic acid
eDNA: extracellular DNA
EDTA: ethylenediaminetetraacetic acid
EPS: extracellular polymeric substances
FLIP7: fly larvae immune peptides 7
GS: gramicidin S
hBD: human β-defensin
LPS: lipopolysaccharide
NO: nitric oxide
PIA: polysaccharide intercellular adhesin
PMB: polymyxin B
QS: quorum sensing
QSIs: quorum sensing inhibitors
RIP: RNA III-inhibiting peptide
1Tb: temporin 1Tb
